# Accounting for cell type hierarchy in evaluating single cell RNA-seq clustering

**DOI:** 10.1186/s13059-020-02027-x

**Published:** 2020-05-25

**Authors:** Zhijin Wu, Hao Wu

**Affiliations:** 1 Department of Biostatistics, Brown University, Providence, 02806 RI USA; 2 Department of Biostatistics and Bioinformatics, Rollins School of Public Health, Emory University, 1518 Clifton Road NE, Atlanta, 30322 GA USA

**Keywords:** Gene expression, Single cell RNA-seq, Clustering

## Abstract

Cell clustering is one of the most common routines in single cell RNA-seq data analyses, for which a number of specialized methods are available. The evaluation of these methods ignores an important biological characteristic that the structure for a population of cells is hierarchical, which could result in misleading evaluation results. In this work, we develop two new metrics that take into account the hierarchical structure of cell types. We illustrate the application of the new metrics in constructed examples as well as several real single cell datasets and show that they provide more biologically plausible results.

## Background

Single cell RNA-sequencing (scRNA-seq) has emerged very recently as a powerful technology to investigate transcriptomic variation and regulation at the individual cell level [1, 2]. Compared with bulk RNA-seq, scRNA-seq reveals cell to cell heterogeneity in transcription, providing critical information to the understanding of biological processes in development, differentiation, and disease etiologies. The technology has gained tremendous interest lately, and many experiments have been conducted to profile different types of complex samples such as cancer [3–5], brain [6, 7], stem cells [8, 9], and immune system [10, 11].

One of the major advantages of scRNA-seq is that it allows the identification of cell types via unsupervised clustering of the transcriptomes from a population of cells. Thus, cell clustering is one of the most common practices and routinely performed in scRNA-seq analysis to identify and discover cell types or subtypes [12]. The development of cell clustering method has been an active research field over the last several years, and a number of methods with software tools have been developed [13–17]. These methods usually partition the cells into several groups, with each group representing a cell type or subtype.

With multiple tools available, comparing their performances becomes a question of interest. To evaluate the performance of a clustering method, the common practice is to compare clustering result with reference labels, where the reference is obtained from another source with high confidence [18]. For example, in some datasets, the cells have been pre-sorted with cell surface markers. In others, known strong cell type-specific gene expression markers could be used to define cell types. The most widely used measures for the agreement between a clustering and a reference label are the adjusted Rand index (ARI) [19] and the normalized mutual information (NMI) [20]. These traditional metrics, however, overlook an important characteristic of single cell data. Unlike many partitioning type of clustering problems, in which the cluster labels are completely exchangeable, the true cluster structure for a cell population is often hierarchical. For example, CD4 T cells and CD8 T cells are both T cells, and T cells and B cells both belong to the more general category “lymphocytes.” Failing to take this true hierarchy into account in the evaluation of clustering results leads to assessments that do not accurately reflect the ability to group cells.

## Results

### Model overview

In this work, we modify the traditional methods and develop two new metrics: weighted Rand index (wRI) and weighted normalized mutual information (wNMI), for the evaluation of cell clustering results from scRNA-seq. The general idea is to obtain weights from cell type hierarchy to reflect different degrees of relationships between cells, and use the weights in RI and MI calculation to reward/penalize the correct/incorrect classification.

Measuring accuracy of supervised clustering is straightforward. Measuring the agreement between two partitions of a population directly is more challenging because in unsupervised clustering, the cluster labels are arbitrary. The number of clusters inferred may not agree with the number of classes in the reference, and a good correspondence between a cluster label and a known class without ambiguity may not exist. The ARI and NMI are two scores developed to indirectly measure the agreement between partitions.

The Rand index (RI) is based on the concordance of pairwise relationships between all pairs of cells, which could be either “within the same group” or “in different groups.” For *n* cells and a total of ${n \choose 2}$ pairwise relationships, the RI computes the proportion of relationships that are in agreement between the clustering and the reference. In other words, for each pair, the relationship defined in the reference is considered either correctly recovered or not. The RI computes the success rate of correctly recovering the relationship, giving all pairwise relationships the same weight. The ARI adjusts the RI by considering the expected value under the null probability model that the clustering is performed randomly given the marginal distributions of cluster sizes. In our proposed wRI, we assign different weights for each pairwise relationship based on the cell type hierarchy information. For example, putting two cells from closely related subtypes (CD4 and CD8 T cells) into one cluster accrues less penalty than grouping cells from more distinct cell types (T cells and B cells). In addition, breaking up a pair of cells of the same type into separate clusters may receive less penalty if cells of that type show higher variation from the mean cell type-specific expression profile, compared to breaking up pairs from a tight cluster.

The mutual information (MI) is a measure of shared “information” between two partitions. It is the proportion of entropy in the reference partition explained by the clustering. Even when the reference knowledge has a hierarchy, the MI ignores the tree structure and only makes use of memberships in the leaf nodes. By definition, there is no entropy among cells within the same leaf node. For a group of cells separated into two cell types, the entropy is the same whether the two cell types are loosely or closely related. In our proposed wNMI, we use a structured entropy that considers the hierarchical relationships between cell types to reflect the accuracy of a clustering algorithm in recovering the cell population’s structure. Detailed description of the wRI and wNMI methods is provided in the “Method and material” section.

### Case studies

#### Constructed examples

**Fig. 1:**
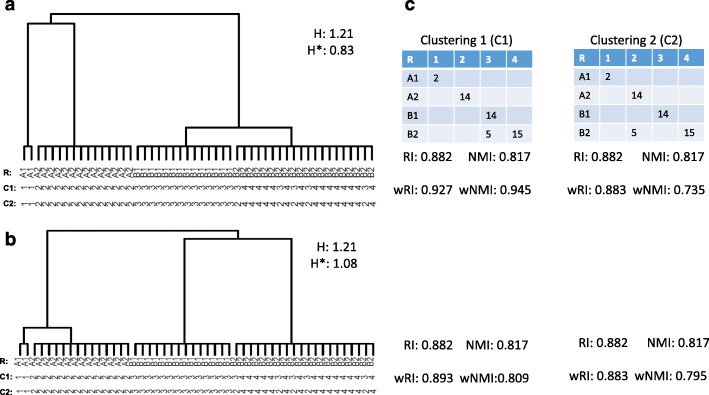
Illustrative examples for using RI/MI and wRI/wMI to evaluate the clustering results. **a**, **b** Two examples of hierarchical relationship between a group of A1, A2, B1, and B2 cells. Texts under the trees indicate cell types from R, reference; C1, clustering 1; and C2, clustering 2. **c** Confusion matrices of two clustering and measures of clustering performance under reference **a** or **b**

We first show constructed toy examples to illustrate the advantages of wRI and wMI in Fig. 1. There are four cell types (represented as A1, A2, B1, and B2) in the true reference with 2, 14, 14, and 20 cells, respectively. We consider two hypothetical tree structures for the cell types, shown as tree A (Fig. 1a) and tree B (Fig. 1b). Two clustering results, both forming four clusters, are compared here. Figure 1c shows the confusion matrices of the clustering results. Clustering 1 (C1) correctly clusters the cells of type A1 and A2, but mistakenly clusters some B2 cells with B1 cells. Clustering 2 (C2) correctly clusters the cells of type A1 and B1, but mistakenly clusters some B2 cells with A2 cells. Intuitively, since B1 and B2 both belong to type B, the mistakes in C1 may be considered more tolerable compared to those in C2, especially when the truth is tree A where B1 and B2 cells are very similar.

The classical metrics (ARI and NMI) give the two clustering results identical scores when the true cell type hierarchy is either tree A or tree B. This is because the classical metrics treat four groups as completely exchangeable, and the two clustering results make the same number of mistakes. In contrast, the new metrics wRI and wNMI depend on the reference structure. In tree A (Fig. 1a) where subtypes B1 and B2 are very closely related, we give lower penalty for mixing these cells. This is reflected by the near perfect (0.927) wRI for C1 and much lower wRI for C2 (0.883). The wNMI also clearly favors C1 over C2 in tree A: 0.945 vs. 0.735. On the other hand, when the true cell hierarchy is tree B (Fig. 1b) where the similarity between B1 and B2 is weaker, the mistakes in C1 (mixing B1 and B2) is nearly as bad as in C2 (mixing B2 with A2), so the advantage of C1 over C2 becomes minimal.

#### Real data application

We apply the new metrics on four public datasets to compare the performances of five popular cell clustering methods, including monocle [13], CIDR [17], Seurat [14], TSCAN [15], and SC3 [16]. Here, we provide brief summaries for these methods. SC3 uses a consensus matrix to summarize *K*-means clustering results over a series of PCA and Laplacian transformed feature matrices, followed by complete-linkage hierarchical clustering. Seurat first selects a set of highly variable genes followed by PCA dimension reduction and then uses a graph-based approach that partitions the cell distance matrix based on the top (usually 10) principal components. It constructs a *K*-nearest neighbor (KNN) graph based on Euclidean distance of the PCs and applies modularity optimization to group cells iteratively. Monocle uses PCA to reduce dimension, often followed by further nonlinear dimension reduction by tSNE or Uniform Manifold Approximation and Projection (UMAP). The clustering is done using density peak clustering or the Louvain algorithm, which is also the default choice for modularity optimization in Seurat. CIDR performs principal coordinate analysis on a dissimilarity matrix between imputed gene expression profiles, where imputation depends on the estimated relationship between dropout rate and gene expression level. The clustering is done on the first few principal coordinates using the R package NbClust. TSCAN first groups genes by hierarchical clustering and reduces individual gene expression to average expression of gene clusters, which are then used to estimate PCs. It then uses model-based clustering (the R package mclust) based on multivariate normal model on the PCs.

The datasets used to evaluate the proposed new metrics are summarized in Table 1. All datasets have known cell types from other experiments, which are used as reference to evaluate clustering results. We obtain the *PBMC1* and *hES* datasets from [18] through the *DuoClustering2018* Bioconductor package. The package provides the true cell type as well as the clustering results from the five methods. The other datasets are obtained from GEO database under accession numbers GSE67835 (*Brain*) and GSE94820 (*PBMC2*).

**Table 1:** A list of datasets used in this work

Dataset	Protocol	No. of cells	No. of cell types	Sample
*PBMC1*	10x	3971	8	PBMC
*hES*	SMARTer	531	9	Human ES
*Brain*	SMARTer	466	9	Human brain
*PBMC2*	SMARTer	1140	5	PBMC

Among the datasets, two are from peripheral blood mononuclear cells (PBMC), but based on different sequencing protocols: 10x Genomics for *PBMC1* [21] and SMARTer sequencing for *PBMC2* [22]. The other two datasets are from human embryonic stem cells (*hES*) [23] and human brain (*Brain*) [24]. The numbers of cells in these datasets range from a few hundred to around 4000. Numbers of cell types range from 5 to 9. These datasets are diverse in terms of tissue types, sequencing protocols, numbers of cells, and cell types, which demonstrate the robustness of the new metrics.

Results for all datasets are shown as Additional file 1: Fig S3–S5. Overall, the values of the proposed metrics tend to be higher than the traditional metrics, as partial credit is given to reasonable but imperfect clustering. However, the increases vary across methods because they make different types of mistakes and all mistakes are not treated equal in our new metrics.

**Fig. 2:**
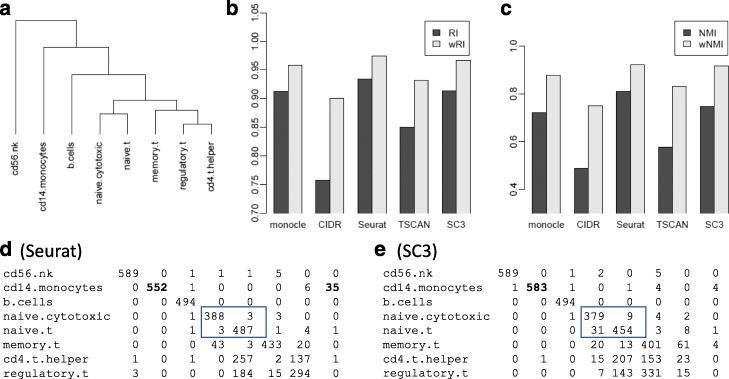
Results from *PBMC1* dataset. **a** The reference hierarchy used in evaluation. **b** RI and wRI for five clustering methods. **c** NMI and wNMI for five clustering methods. **d** Confusion matrix for Seurat. **e** Confusion matrix for SC3

In particular, Fig. 2 shows the results from the *PBMC1* dataset, which was generated by the 10x Genomics GemCode protocol to profile the transcriptome of eight pre-sorted cell types (B cells, naive cytotoxic T cells, CD14 monocytes, regulatory T cells, CD56 natural killer cells, memory T cells, CD4 T helper cells, and naive T cells) in peripheral blood mononuclear cells (PBMC). Figure 2a shows the true hierarchical structure of the eight cell types, constructed based on the average gene expression profiles (details in the “Method and material” section). Figure 2b, c shows the values of unweighted and weighted RI and NMI from the five clustering methods. All five methods show better performance under the new metrics, but with different performance gains. CIDR and TSCAN show more substantial gains under the new metrics, indicating that their performances are not as bad as suggested by RI and NMI. Seurat appears to be substantially better than SC3 based on RI and NMI. From the new metrics, the differences between them become much smaller.

We include the confusion matrices for Seurat and SC3 (Fig. 2d, e) to provide more insight. Both methods do a near perfect job in identifying CD56 natural killer cells and B cells as distinct clusters. Both face difficulty distinguishing regulatory T, CD4 T helper, and memory T cells. Seurat appears to separate naive cytotoxic and naive T cells better (rectangles in panels d and e), which would give Seurat an advantage in RI and NMI. But confusing these cells are not penalized as much in the new metric given their close similarity. On the other hand, SC3 does a much better job in identifying CD14 monocytes by keeping nearly all of them in one distinct cluster. The overall performance of these methods is similar, as reflected by the new metrics that take the cell type hierarchy into consideration. To summarize, the new metrics, by considering the cell type hierarchy, provide a more objective evaluation of the clustering results from different methods.

## Discussions

In conclusion, we propose two new metrics for evaluating clustering results from scRNA-seq data. The essence of the metrics is to take into account the hierarchy in cell type relationships. These hierarchical relationships are often at least partially known, based on existing knowledge on cell lineage and cell-proliferative hierarchies [25–27]. Ignoring the information in the hierarchy may result in biased and misleading assessment of cell clustering result. The proposed adjusted metrics capture the hierarchical structure in the reference cell types, which overcome the drawback and provide more biological relevant measures of the clustering performance. The proposed methods for computing the new metrics are implemented as an R package available at https://github.com/haowulab/Wind [28].

The comparisons we present in this manuscript are used to illustrate the new metrics and not meant to advocate one clustering method over another. Scientists may choose various tuning parameters according to each method, or use various strategies to filter genes and to reduce dimensions before clustering, or use different strategies to choose the number of clusters. All of these choices will affect the evaluation of clustering performance, whether one uses traditional metrics or the proposed ones.

The interpretation of the wRI is the agreement in cell grouping between the clustering and the hierarchical reference. The interpretation of the wNMI is the hierarchical heterogeneity (entropy) of the cells explained by the clustering. Both, of course, depend on the reference hierarchical structure. There is not necessarily a consensus of the hierarchical tree of known branch lengths for the cell types under study. In some situations, the tree topology may be known but not the branch lengths, such as some cell lineage relationships [29, 30]. This problem will be at least partially relieved as single cell data continue to accumulate rapidly in the public domain, increasing our ability to construct accurate hierarchical relationships between cell types [31]. Nevertheless, it is critical that the weight matrices are chosen independent of the development and/or evaluation of clustering methods. Otherwise, we could face issues similar to p-hacking [32]: a user could try a large number of weighting schemes until a favored method appears to show optimal result. Many scientists have advocated for pre-registration [33] to promote transparency and reproducibility. Pre-registration should include both analysis plan and evaluation plan. Sensitivity analysis also helps to determine how robust the weighted metrics are to the choices of weight matrices. For that, we include some sensitivity analysis in Additional file 1: Section S3.

Though motivated by scRNA-seq data, the new metrics are relevant in many other applications. For example, in the clustering of individuals of different species, we have phylogenetic structure as reference. The reference not only separates different species but also provides a hierarchical structure that allows one to score the grouping of individuals from two closely related species (a chimpanzee and a human) with lower penalty than grouping individuals that diverged much earlier (a lemur and a human). In some other applications, the weights can be chosen to reflect how we value the ability of separating certain types, such as in clustering chemical compounds [34].

## Method and material

### The weighted Rand index

Consider a population of *n* cells with reference cell types and a clustering result. The reference is treated as gold standard, and we want to evaluate the agreement between the clustering and the reference. For cell *k*, the reference *R* is a mapping $R(k):\{1,2,\dots, n\} \rightarrow \{1,2,\dots, J\}$. The clustering provides another partition with $C(k) \in \{1,2,\dots, I\}$, where *I* may or may not coincide with *J*.

The Rand index considers pairwise relationships between any two cells in a population. For a pair of cells indexed by *k*_1_,*k*_2_ (1≤*k*_1_<*k*_2_≤*n*), the pairwise relationship is in agreement if ***1***{*C*(*k*_1_)=*C*(*k*_2_)}=***1***{*R*(*k*_1_)=*R*(*k*_2_)}, where ***1*** is the indicator function. If we consider cells within a cluster as “related” and cells in different cluster as “separated,” the pairwise relationship between two sets of partitions can be stratified in a 2×2 contingency table shown in Table 2. Here, rows are defined based on the reference (R), where two cells are deemed “related” when ***1***{*R*(*k*_1_)=*R*(*k*_2_)}=1, and “separated” otherwise. Columns are based on the clustering results (C), where ***1***{*R*(*k*_1_)=*R*(*k*_2_)}=1 means two cells are “related.”

**Table 2:** Agreement of pairwise relationship between reference (R) and clustering results (C)

	C		
R	Related	Separated	
Related	*N*_11_	*N*_10_	*N*_1+_
Separated	*N*_01_	*N*_00_	*N*_0+_
	*N*_+1_	*N*_+0_	*N*

Out of $N={n \choose 2}=N_{11}+N_{10}+N_{01}+N_{11}$ pairs, there are *N*_11_+*N*_00_ concordant pairs between the clustering and the reference. The Rand index (RI) is defined as the proportion (*N*_11_+*N*_00_)/*N*. To adjust for random chance, the RI can be modified by taking into account the expected number of pairing agreements. This definition of RI considers the pairwise relationship inferred in the clustering result as either concordant with the reference or not. Formally speaking, it first defines a score for a pair of cells *k*_1_ and *k*_2_: *s*(*k*_1_,*k*_2_;*C*,*R*)=1 if ***1***{*C*(*k*_1_)=*C*(*k*_2_)}=***1***{*R*(*k*_1_)=*R*(*k*_2_)}, and 0 otherwise. Then, the overall agreement score between *C* and *R* is defined as:

$$S(C,R)= \sum_{1\leq k_{1}< k_{2}\leq n} s(k_{1},k_{2};C,R).$$

It is easy to verify that $S(R,R)= \sum _{1\leq k_{1}< k_{2}\leq n} 1= {n\choose 2}$. The RI is defined as *RI*(*C*,*R*)=*S*(*C*,*R*)/*S*(*R*,*R*).

This RI definition is sensible when all cell types are equivalent and lack a hierarchical structure. In reality, cell types in the reference have a hierarchical structure, which includes cell types that may be a subcategory of others. For example, there are more general terms like lymphocytes and more specific cell types including T cells and B cells, and the T cells can be further categorized by cell surface markers (e.g., CD4 T cells and CD8 T cells). Thus, in scRNA-seq clustering, the groups in the reference are no longer exchangeable. To reflect the potentially nested relationships between these cell types, we introduce a weighting scheme that allows the importance of pairwise relationships to vary.

To account for the intrinsic cell type hierarchy and characteristics, we redefine the agreement score as:

$$S^{*}(C,R)=\sum_{1\leq k_{1}< k_{2}\leq n} s^{*}(k_{1},k_{2}; C, R),$$

where

$$s^{*}(k_{1},k_{2}; C, R)=\left\{ \begin{array}{lr} \boldsymbol{W}^{1}_{i,j} &\text{if}\ C(k_{1})=C(k_{2})\\ \boldsymbol{W}^{0}_{i,j} &\text{if}\ C(k_{1})\neq C(k_{2})\\ \end{array}\right. $$

Here, *i* and *j* are the cell type indexes based on the reference: *i*=*R*(*k*_1_),*j*=*R*(*k*_2_). The proposed weighted RI (wRI) is then defined as *wRI*(*C*,*R*)=*S*^∗^(*C*,*R*)/*S*^∗^(*R*,*R*).

Both ***W***^1^ and ***W***^0^ are *J*×*J* weight matrices representing one’s willingness to reward/penalize the correct/incorrect pairwise relationships. The entry (*i*,*j*) in ***W***^1^ is the score for putting two cells of type *i* and *j* in the same cluster. When two cells are put in the same cluster, i.e., *C*(*k*_1_)=*C*(*k*_2_), the classical RI gives a score 1 if *R*(*k*_1_)=*R*(*k*_2_), and a score 0 if *R*(*k*_1_)≠*R*(*k*_2_). When the reference has a hierarchical structure, however, we may wish to treat “mistakes,” i.e., *R*(*k*_1_)≠*R*(*k*_2_), differently and give partial credit if *R*(*k*_1_) is a cell type close to *R*(*k*_2_). For example, clustering cells from different but closely related cell types (CD4 T cell and CD8 T cell) is not penalized as much as clustering cells from completely unrelated cell types.

The entry (*i*,*j*) in ***W***^0^ is the score for separating two cells of type *i* and *j* in different clusters. When two cells are separated into different clusters, i.e., *C*(*k*_1_)≠*C*(*k*_2_), the classical RI has binary scores *S*(*k*_1_,*k*_2_)=1 if *R*(*k*_1_)≠*R*(*k*_2_) (correctly keeping cells *k*_1_ and *k*_2_ in separate groups), and 0 otherwise. Depending on how homogeneous cells are within a certain cell type, we may also treat the “importance” of keeping cells in the same cluster differently. When all cells of the same type are considered identical, and all cell types form tight clusters, the traditional RI definition is reasonable. But when some cell types consist of more diverse cells, thus potentially contain subtypes, we could use weights to reflect the allowance for breaking up a pair depending on the differences in tightness.

#### Obtaining the weights for wRI

The weight matrices ***W***^1^ and ***W***^0^ can be specified by users based on prior biological knowledge or estimated from data based on gene expression values. Note that choosing ***W***^1^ as the identity matrix *I*_*J*×*J*_, and ***W***^0^=1−***W***^1^ reduces *S*^∗^ to *S*, as used in classical RI. Here, we describe our recommended strategies for obtaining the weight matrices.

*Using prior knowledge or data.* We let ***W***^1^ has diagonal values 1 and off-diagonal less than 1, reflecting that recovering a tie in the reference receives full credit, but forming new ties may receive partial credit. The ***W***^1^ matrix reflects the similarity between reference cell types. For some well-studied cell populations, prior biological knowledge about cell type hierarchy exists. For example, the lineage of blood cells from hematopoietic cells is studied extensively. The similarity matrix between cell types used to construct cell lineage trees based on genomic variability [35] may be used as the weights *W*^1^ here. We may also establish similarity using gene expression profiles from public data depositories. A natural way is to compute mean expression profiles from each cell type either by using pure bulk data or by averaging labeled single cell data, and use similarity measures such as Pearson’s or Spearman’s correlation.

We make ***W***^0^ has off-diagonal values 1 and diagonal values between 0 and 1, reflecting that keeping the separation existing in the reference receives full credit, but breaking a (weak) tie may not reduce the score completely to 0. The diagonal values in ***W***^0^ reflect the penalty for splitting cells from the same type into different clusters: 0 reflects full penalty and a value between 0 and 1 reflects the tolerance for splitting a pair. We may allow more tolerance for splitting cell types with greater heterogeneity, which could be assessed by inter-cellular variance within a cell type. With rapidly increasing single cell data including, but not limited to, DNA methylation landscape, chromatin accessibility, and transcription, we may choose any of these sources to establish different heterogeneity within cell types. For example, we may represent the heterogeneity as the average distance between individual cell profiles to the mean profile of its cell type.

*Using the scRNA-seq data used in clustering.* Using the traditional RI in evaluation requires the reference of the true cell types only. To compute wRI, we also need the weight matrices. When we do not have prior knowledge or external data, we may establish reasonable weights using the scRNA-seq data that the clustering is performed on. Using the reference cell type labels, we obtain mean expression profiles for each cell type. If multiple batches are involved and batch effects are suspected, the mean expression profiles should be computed after batch effects are removed [36–38]. Again, we set the diagonal of ***W***^1^ at 1. We may compute the off-diagonal values of ***W***^1^ using a similarity measure (such as Pearson’s correlation coefficient *r*) of the mean expression profiles between cell types. We set the off-diagonal values of ***W***^0^ at 1 and use a heterogeneity measure between 0 and 1 for each cell type for the diagonal values. For example, the heterogeneity measure could be the average dissimilarity (such as 1−*r*) between each individual cell’s expression profile and its cell type mean profile.

For the illustration results presented in this manuscript, we estimate the weights from the same dataset that the clustering is performed on. Specifically, we compute the mean expression profiles of each cell type and selected the top 1000 genes with the greatest variance in log expression. We set $\boldsymbol {W}^{1}_{i,j}$ as the Pearson correlation of the mean expression of these genes in cell types *i* and *j*. We set off-diagonal values of ***W***^0^ to 1 and compute $\boldsymbol {W}^{0}_{i,i}$ based on the inter-cellular expression variances within cell type *i*. To be specific, we take expressions for all cells in cell type *i* and compute their pairwise Pearson’s correlation coefficients. For cell type containing *n* cells, there are ${n \choose 2}$ correlation coefficients. We compute the average of these correlations and define $\boldsymbol {W}^{0}_{i,i}$ as $1-\sum _{1\leq k< l\leq n} r_{k,l}/{n\choose 2}$. Sensitivity to these choices is discussed in Additional Files 1: Section S3.

#### Further interpretation for RI and wRI

The Rand index values the agreement between the reference and the clustering in both the “related” (same cluster) and “separated” (different cluster) relationships. The RI is the proportion of correctly identified relationships out of the total ${n \choose 2}$ pairs. We can also view this as a weighted average of the two accuracies:

$$\begin{array}{*{20}l} RI &=(N_{11}+N_{00})/N=\frac{N_{11}}{N_{+1}} \frac{N_{+1}}{N}+\frac{N_{00}}{N_{+0}}\frac{N_{+0}}{N}  \\ &= \frac{N_{11}}{N_{+1}} w+\frac{N_{00}}{N_{+0}}(1-w)  \end{array} $$

Here, *N*_11_/*N*_+1_ is the proportion of true “related” relationships among those identified in the clustering, and *N*_00_/*N*_+0_ is the proportion of true “separated” relationships among those identified. In other words, if the clustering is meant to clearly identify relationships among pairs of subjects and we consider pairs placed in the same cluster as a “positive” result, *N*_11_/*N*_+1_ is the positive predictive value (PPV), and *N*_00_/*N*_+0_ is the negative predictive value (NPV). The RI is an average of these two predicative values, with weights proportional to the split of positives and negatives. In some comparisons, we may want to consider the two predictive values directly instead of reducing these to a simple weighted average. The predictive values while considering hierarchical structures become:

$$S^{*}_{1}(C,R)= \frac{1}{N_{+1}} \sum_{1\leq k_{1}< k_{2}\leq n} s^{*}(k_{1},k_{2}; C, R) \boldsymbol{1}\{C(k_{1})=C(k_{2})\}.$$

and

$$S^{*}_{0}(C,R)= \frac{1}{N_{+0}} \sum_{1\leq k_{1}< k_{2}\leq n} s^{*}(k_{1},k_{2}; C, R) \boldsymbol{1}\{C(k_{1})\neq C(k_{2})\}$$

We provide these two weighted predictive values in the software package to assist the interpretation of the clustering performance. As noted earlier, in the simple cases when ***W***^1^=*I*_*J*×*J*_ and ***W***^0^=1−***W***^1^, these values reduce to the original forms $\frac {N_{11}}{N_{+1}}$ and $\frac {N_{00}}{N_{+0}}$.

### The weighted mutual information

Another way to compare the agreement between a clustering result and the reference is the mutual information (MI). The MI measures how much information in one grouping is explained/captured by another grouping. In the case of a gold reference that partitions the population of *n* cells into *J* classes, we denote $R=\{r_{1},r_{2},\dots,r_{J}\}$, where the *r*_*j*_’s are mutually exclusive sets of cells with ∪_*j*_*r*_*j*_ be the complete population of cells. The clustering algorithm result being evaluated here gives *I* clusters represented as $C=\{c_{1},\dots,c_{I}\}$. One can tabulate the number of cells between reference and clustering result in a contingency table shown as Table 3.

**Table 3:** Cell membership agreement between reference (R) and clustering results (C)

	C						
R	1	2	$\dots $	*i*	$\dots $	I	
1	*n*_11_	*n*_12_		*n*_1*i*_		*n*_1*I*_	*n*_1+_
2	*n*_21_	*n*_22_		*n*_2*i*_		*n*_2*I*_	*n*_2+_
⋯	⋯	⋯		⋯		⋯	⋯
*j*	*n*_*j*1_	*n*_*j*2_		*n*_*ji*_		*n*_*jI*_	*n*_*j*+_
⋯	⋯	⋯		⋯		⋯	⋯
*J*	*n*_*J*1_	*n*_*J*2_		*n*_*Ji*_		*n*_*JI*_	*n*_*J*+_
	*n*_+1_	*n*_+2_		*n*_+*i*_		*n*_+*I*_	*n*

Mutual information is defined as:

$$\begin{array}{@{}rcl@{}} I(C;R) &=& \sum_{j}\sum_{i} P(r_{j}\cap c_{i})\log\frac{P(r_{j}\cap c_{i})}{P(r_{j})P(c_{i})} \\ &=& \sum_{j}\sum_{i} \frac{|r_{j}\cap c_{i}|}{n} \log\frac{n |r_{j}\cap c_{i}|}{|r_{j}|| c_{i}|} \\ &=& \sum_{j}\sum_{i} \frac{n_{ji}}{n}\log\frac{n n_{ji}}{n_{j+} n_{+i}} \end{array} $$

For each non-zero entry in the *J*×*I* table, the mutual information has value *p*_*ji*_∗ log(Observed/Expected) where *p*_*ji*_ is the proportion of “Type *j*” cells in the *i*th cluster. In a perfect clustering, *I*=*J* and we will be able to get a *J*×*J* table such that each row and each column has one and only one non-zero entry. We can rearrange this table such that the non-zero entry is on the diagonal with values *n*_*jj*_=|*r*_*j*_|=*n*_*j*+_. In this perfect case, the mutual information is the same as the entropy of *R* itself, which is defined as:

$$\begin{array}{@{}rcl@{}} H(R)&=&-\sum_{j} P(r_{j})\log P(r_{j})\\ &=& \sum_{j} \frac{|r_{j}|}{n}\log\frac{|r_{j}|}{n}=\sum_{j} \frac{n_{j+}}{n}\log\frac{n_{j+}}{n} \end{array} $$

One interpretation of the mutual information is via the conditional entropy, since the MI can also be defined as *MI*(*C*,*R*)=*H*(*R*)−*H*(*R*|*C*), where *H*(*R*|*C*) is the conditional entropy of *R* given *C*. If the clustering result perfectly recovers the grouping in the reference, the conditional entropy *H*(*R*|*C*)=0, and then, *MI*(*C*,*R*)=*H*(*R*). Thus, the MI can be seen as the entropy of the true classes in the reference that can be explained by the clustering. The MI is often turned into “normalized mutual information” (NMI) by dividing by either the arithmetic or geometric mean of *H*(*R*) and *H*(*C*), thus having a value between 0 and 1. We can also see that:

$$\begin{array}{*{20}l} NMI (C, R)&=\frac{MI(C, R)}{[H(R)+H(C)]/2}  \\ &=\frac{H(R)-H(R|C)}{H(R)}\frac{H(R)}{[H(R)+H(C)]/2} \end{array} $$

Here, the first factor on the right-hand side represents the amount of entropy in the reference explained by the clustering, similar to the *R*^2^ in linear regression models (where the variance, instead of the entropy, is explained by a linear model). The second factor weighs the relative complexity of the reference and the clustering to balance: dividing the population into too many clusters will increase the *R*^2^-like factor but will decrease the second factor. Trivial overfitting can make each cell as a singleton cluster and achieve a MI as high as *H*(*R*), but is of little use. A good clustering is a partition that can recover most of the structure without breaking into too many groups.

Now, suppose the true reference has a hierarchical structure represented by a dendrogram. The finest level contains *J* cell types, but we could trim the dendrogram/tree at higher levels and have 2 to *J*−1 number of classes. Let *R*_*j*_ denote the clustering resulted from cutting the tree to form *j* groups. The total entropy could be divided into stepwise entropy as:

$$H(R_{J})=H(R_{1})+H(R_{2}|R_{1})+H(R_{3}|R_{2})+\dots +H(R_{J}|R_{J-1}).$$

Obviously, when all cells are considered as one type, *H*(*R*_1_)=0.

When all *J* classes are distinct, each conditional entropy has the same weight and the total entropy *H*(*R*_*J*_) is the simple summation (similar to the example given in Additional files 1: Fig. S2A). When the classes have a hierarchical structure, however, the entropy in the classical definition does not reflect the true complexity or information contained in the population of cells. We introduce the “structured entropy” by weighting the stepwise conditional entropy:

$$\begin{array}{*{20}l} H^{*}(R)=H(R_{1})&+d_{1}H(R_{2}|R_{1})+d_{2}H(R_{3}|R_{2})+\dots  \\ &+d_{J-1}H(R_{J}|R_{J-1})  \end{array} $$

Here, *d*_*j*_ is the distance in the dendrogram representing the level of separation from *j* groups to *j*+1 groups, as illustrated in Additional file 1: Fig. S1. The modified conditional entropy given the clustering C is:

$$\begin{array}{*{20}l} H^{*}(R|C)=H(R_{1}|C) &+d_{1}H(R_{2}|R_{1},C)+d_{2}H(R_{3}|R_{2},C)+\dots  \\ &+d_{J-1}H(R_{J}|R_{J-1},C)  \end{array} $$

Given this modified entropy, we can then define the weighted mutual information as:

$$wMI(C,R)=H^{*}(R)-H^{*}(R|C), $$

and the weighted normalized mutual information as:

$$wNMI(C,R)=\frac{wMI(C,R)}{H^{*}(R)}\frac{H(R)}{[H(R)+H(C)]/2}$$

#### Obtaining the weights for WNMI

The full hierarchical information needed to compute the structured entropy includes the topology of the dendrogram as well as the length of the branches. The topology is often known, including blood cell types and many cell types in the nervous system. The *d* values may be obtained using distance measures between cell types, either using existing gene expression data from pure bulk expression or average expression profile from single cell data. When the topology of the hierarchical structure is also lacking, we may use hierarchical clustering on cell type expression profiles either from bulk data or by averaging single cell data. As in obtaining weights for wRI, when multiple batches are involved, the mean expression profiles should be computed after batch effects removal [36–38].

The weights used in the examples in this manuscript is computed based on the heights of branches of the hierarchical tree of different cell types. We first compute mean expression profiles for all cell types and select 1000 marker genes with the greatest between cell type variation. A hierarchical tree is then constructed based on the mean expression from marker genes, using the hclust function in R. We obtain the tree height (from the bottom) at each branching point and standardize by dividing the maximum tree height.

## Supplementary information

**Additional file 1** Supplemental Materials of “Accounting for cell-type hierarchy in evaluating single cell RNA-seq clustering.”

**Additional file 2** Review history.

## Data Availability

The implementation of the method presented is available at https://github.com/haowulab/Wind as an open source software under GPL license [28]. Several publicly available datasets are used in this manuscript, as summarized in Table 1. The PBMC1 dataset is described in [21], and the hES dataset is described in [23]. These two datasets are conveniently provided by *DuoClustering2018* Bioconductor package [18]. The *Brain* dataset [24] and the *PBMC2* dataset are available from the Gene Expression Omnibus database under accession numbers GSE67835 and GSE94820, respectively.
