# Supplementary material for: Accounting for cell type hierarchy in evaluating single cell RNA-seq clustering
Source: Genome Biol. 2020 May 25;21:123. doi: 10.1186/s13059-020-02027-x (PMC7249323; doi:10.1186/s13059-020-02027-x)
Supplement: Supplementary file 1 — Additional file 1 Supplemental Materials of “Accounting for cell-type hierarchy in evaluating single cell RNA-seq clustering.” [file 13059_2020_2027_MOESM1_ESM.pdf]

Supplemental Materials of "*Accounting for cell-type hierarchy in evaluating single cell RNA-seq clustering*"

S1 Illustration of stepwise entropy

When a hierarchical tree with J leafs is cut at various levels in to 2 to J branches, each cut forms a partial partition. Let R_J represent the final partition and R_j represent the partition from the cut associated with j clusters. The total entropy for R_J, $H(R_J)$, may be computed as a summation of stepwise conditional entropy:

$$H(R_J) = H(R_1) + H(R_2|R_1) + H(R_3|R_2) + \ldots + H(R_J|R_{J-1})$$

The structured entropy that reflects the separation of the branches is defined as

$$H^*(R_J) = H(R_1) + d_1 H(R_2|R_1) + d_2 H(R_3|R_2) + \ldots + d_{J-1} H(R_J|R_{J-1})$$

Obviously, when all cells are considered as one type, $H(R_1) = 0$. When all J types are considered distinct, defining $d_j = 1 \forall j$ reduces H^* to H. However, smaller d values for the lower branches give lower weight to the details of the tree compared to higher weights for the main structure of the tree. Two trees with the same topology but different branch lengths can lead to different structured entropy H^* values.

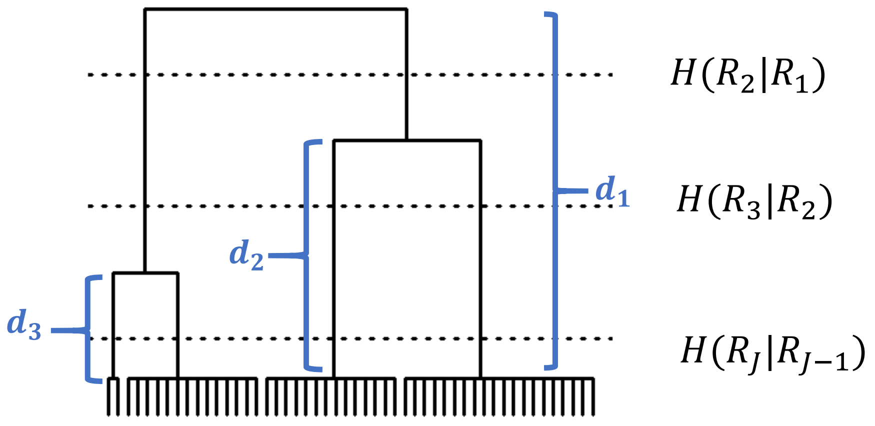

Fig. S1: Illustration of stepwise entropy

Figure S2 below illustrates two extreme cases. Figure S2A represents four clusters that are essentially distinct and all d close to 1, thus the H^* is approximately the same as the classical entropy. Figure S2A represents two large classes each with two sub group that are very similar, in which case the weighted entropy of four classes is only slightly greater than the entropy of dividing into two large classes (and ignoring the entropy within sub groups).

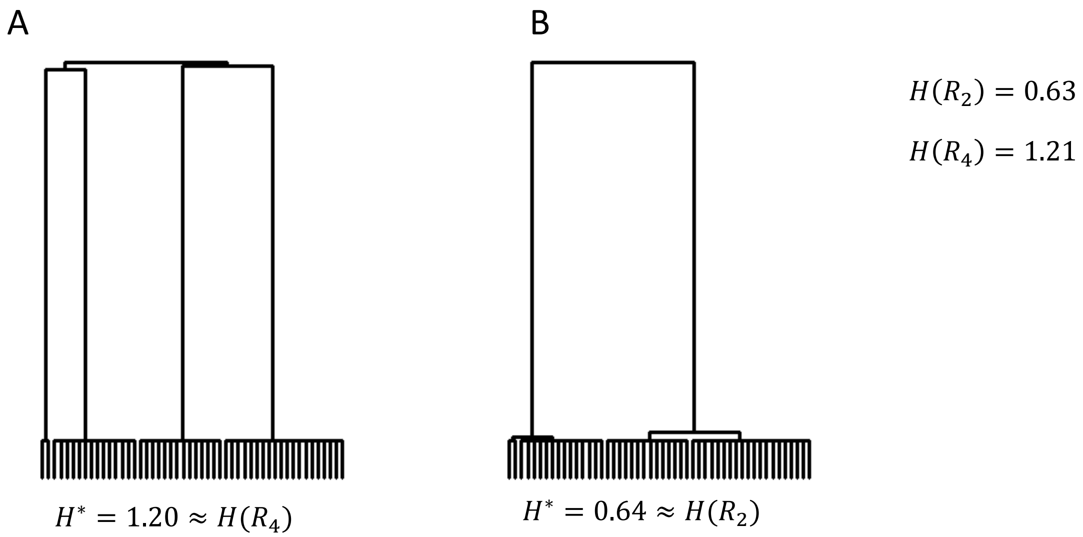

Fig. S2: Illustration of weighted entropy. A: An example of four distinct classes and $d_1 \approx d_2 \approx d_3 \approx 1$, thus $H^* \approx H(R_4)$ B: An example where $d_1 \approx 1$ and $d_2 \approx d_3 \approx 0$, thus $H^* \approx H(R_4)$

S2 Additional Results for evaluating clustering methods

S2.1 Results from hES, Brain and PBMC2 data

Figures below show the results for reanalyzing the three additional datasets listed in Table 1. Each figure contains three panels for (A) the hierarchical structure of the cell types; (B) Comparison of Rand index and weighted Rand index from five clustering methods; and (C) Comparison of normalized mutual information and weighted normalized mutual information from five clustering methods.

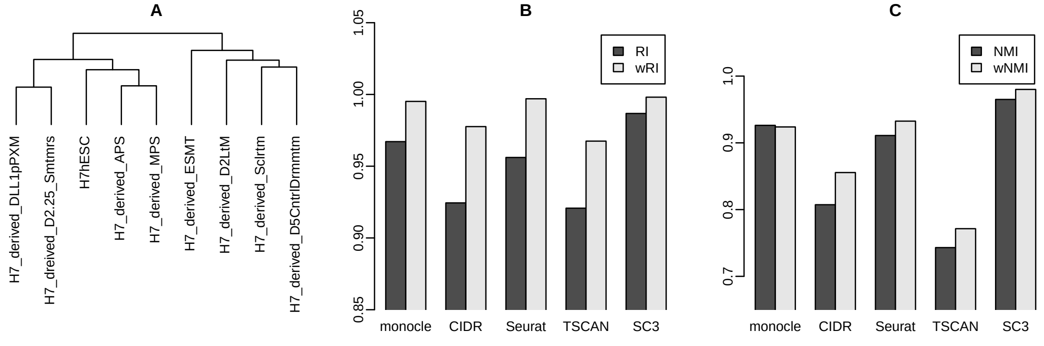

Fig. S3: Results from the *hES* data

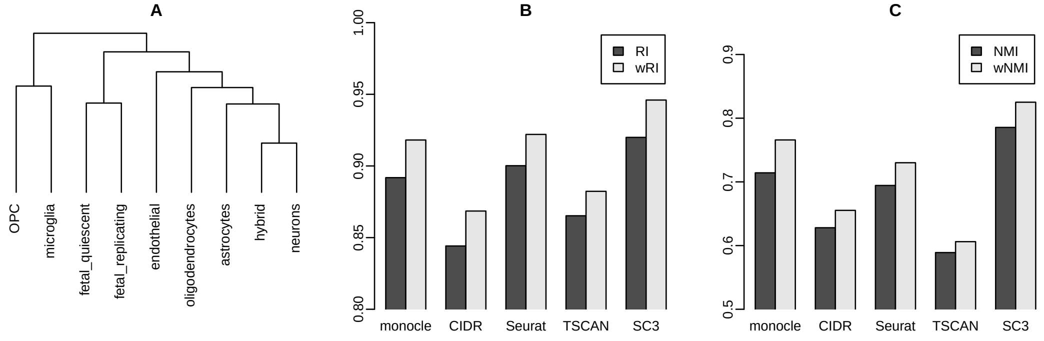

Fig. S4: Results from the *Brain* data

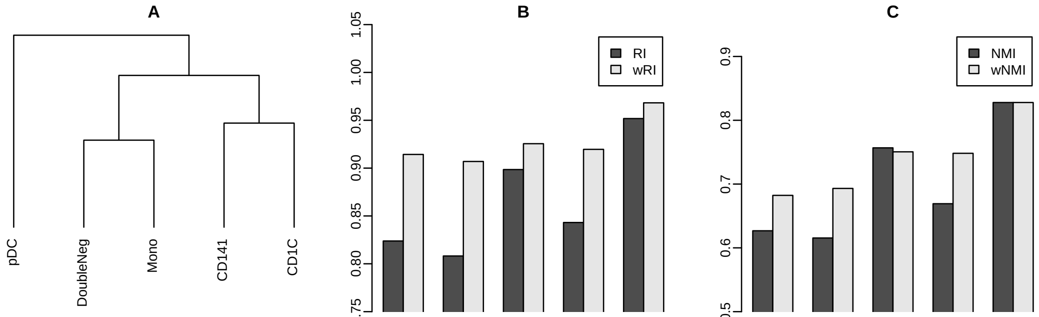

Fig. S5: Results from *PBMC2* data

S2.2 Confusion matrices from *hES* data

	1	2	3	4	5	6	7	8	9
H7_derived_DLL1pPXM	74	0	0	0	0	0	0	0	0
H7_dreived_D2.25_Smtmrs	0	85	0	0	0	0	0	0	0
H7hESC	0	0	53	0	0	0	0	2	0
H7_derived_APS	0	0	0	62	0	0	0	0	0
H7_derived_MPS	0	0	0	0	22	0	0	0	0
H7_derived_ESMT	0	0	0	0	0	43	0	0	0
H7_derived_D2LtM	0	0	0	2	0	0	53	0	0
H7_derived_Sclrtm	0	0	0	6	0	0	62	0	0
H7_derived_D5CntrlDrmmtm	0	0	0	0	0	0	1	1	65

Table S1: hES data, monocle

	1	2	3	4	5	6	7	8	9
H7_derived_DLL1pPXM	72	2	0	0	0	0	0	0	0
H7_dreived_D2.25_Smtmrs	75	10	0	0	0	0	0	0	0
H7hESC	0	0	51	0	4	0	0	0	0
H7_derived_APS	0	0	0	62	0	0	0	0	0
H7_derived_MPS	0	0	0	8	14	0	0	0	0
H7_derived_ESMT	0	43	0	0	0	0	0	0	0
H7_derived_D2LtM	0	0	0	0	1	0	54	0	0
H7_derived_Sclrtm	0	0	0	1	6	11	0	46	4
H7_derived_D5CntrlDrmmtm	0	0	0	0	0	15	0	4	48

Table S2: hES data, CIDR

	1	2	3	4	5	6	7
H7_derived_DLL1pPXM	74	0	0	0	0	0	0
H7_dreived_D2.25_Smtmrs	0	85	0	0	0	0	0
H7hESC	0	0	55	0	0	0	0
H7_derived_APS	0	0	0	62	0	0	0
H7_derived_MPS	0	0	0	22	0	0	0
H7_derived_ESMT	0	0	0	0	0	41	2
H7_derived_D2LtM	0	0	0	0	0	0	55
H7_derived_Sclrtm	0	0	0	2	0	0	66
H7_derived_D5CntrlDrmmtm	0	0	0	0	64	0	3

Table S3: hES data, Seurat

	1	2	3	4	5	6	7	8	9
H7_derived_DLL1pPXM	57	13	0	0	0	0	0	0	4
H7_dreived_D2.25_Smtmrs	1	78	0	0	0	6	0	0	0
H7hESC	0	0	33	16	2	0	1	0	3
H7_derived_APS	0	0	56	3	3	0	0	0	0
H7_derived_MPS	0	0	2	2	18	0	0	0	0
H7_derived_ESMT	0	2	0	0	0	39	0	0	2
H7_derived_D2LtM	0	0	0	0	3	0	50	0	2
H7_derived_Sclrtm	0	0	0	2	0	0	0	43	23
H7_derived_D5CntrlDrmmtm	0	0	0	0	0	0	0	5	62

Table S4: hES data, TSCAN

	1	2	3	4	5	6	7	8	9
H7_derived_DLL1pPXM	74	0	0	0	0	0	0	0	0
H7_dreived_D2.25_Smtmrs	0	85	0	0	0	0	0	0	0
H7hESC	0	0	55	0	0	0	0	0	0
H7_derived_APS	0	0	0	62	0	0	0	0	0
H7_derived_MPS	0	0	0	22	0	0	0	0	0
H7_derived_ESMT	0	0	0	0	0	43	0	0	0
H7_derived_D2LtM	0	0	0	0	1	0	54	0	0
H7_derived_Sclrtm	0	0	0	3	0	0	0	65	0
H7_derived_D5CntrlDrmmtm	0	0	0	0	0	0	0	0	67

Table S5: hES data, SC3

S2.3 Confusion matrices from *Brain* data

	1	2	3	4	5	6	7	8
OPC	14	4	0	0	0	0	0	0
microglia	1	14	0	0	1	0	0	0
fetal_quiescent	0	0	89	19	0	0	0	2
fetal_replicating	0	0	0	25	0	0	0	0
endothelial	0	16	0	0	3	1	0	0
oligodendrocytes	2	0	0	0	33	3	0	0
astrocytes	0	0	0	0	0	0	62	0
hybrid	6	0	0	0	2	29	2	7
neurons	4	0	0	0	0	91	3	33

Table S6: Brain data, monocle

	1	2	3	4	5	6	7	8	9
OPC	13	4	0	1	0	0	0	0	0
microglia	1	14	0	0	0	1	0	0	0
fetal_quiescent	16	0	70	0	0	0	0	0	24
fetal_replicating	4	0	1	20	0	0	0	0	0
endothelial	0	19	0	0	1	0	0	0	0
oligodendrocytes	2	0	0	0	1	34	0	1	0
astrocytes	9	0	0	0	0	0	53	0	0
hybrid	21	1	0	0	17	2	1	4	0
neurons	51	0	0	0	64	0	0	16	0

Table S7: Brain data, CIDR

	1	2	3	4	5	6	7
OPC	14	4	0	0	0	0	0
microglia	1	14	0	0	1	0	0
fetal_quiescent	0	0	110	0	0	0	0
fetal_replicating	18	0	7	0	0	0	0
endothelial	17	0	0	0	3	0	0
oligodendrocytes	1	0	0	0	36	1	0
astrocytes	3	0	0	1	1	0	57
hybrid	9	2	0	11	2	12	10
neurons	1	1	0	52	0	76	1

Table S8: Brain data, Seurat

	1	2	3	4	5	6	7	8	9
OPC	8	0	0	3	7	0	0	0	0
microglia	14	0	0	1	1	0	0	0	0
fetal_quiescent	1	47	62	0	0	0	0	0	0
fetal_replicating	12	3	4	5	1	0	0	0	0
endothelial	10	0	0	3	6	1	0	0	0
oligodendrocytes	1	0	0	28	6	2	0	1	0
astrocytes	10	0	0	1	0	0	49	2	0
hybrid	1	1	0	3	1	22	2	12	4
neurons	0	1	1	0	0	14	1	50	64

Table S9: Brain data, TSCAN

	1	2	3	4	5	6	7	8	9
OPC	14	4	0	0	0	0	0	0	0
microglia	1	14	0	0	0	1	0	0	0
fetal_quiescent	0	0	109	0	1	0	0	0	0
fetal_replicating	1	0	0	20	4	0	0	0	0
endothelial	0	20	0	0	0	0	0	0	0
oligodendrocytes	2	0	0	0	0	35	0	1	0
astrocytes	0	0	0	0	0	1	60	1	0
hybrid	6	1	0	0	0	2	1	35	1
neurons	2	1	0	0	1	0	0	105	22

Table S10: Brain data, SC3

S2.4 Confusion matrices from *PBMC2* data

	1	2	3	4
pDC	180	0	1	11
DoubleNeg	12	84	84	12
Mono	44	0	327	1
CD141	3	0	0	189
CD1C	4	0	0	188

Table S11: PBMC2 data, monocle

	1	2	3	4	5
pDC	174	1	0	7	10
DoubleNeg	1	175	0	11	5
Mono	1	157	171	1	42
CD141	0	2	1	186	3
CD1C	0	9	0	179	4

Table S12: PBMC2 data, CIDR

	1	2	3	4	5	6	7	8	9	10
pDC	172	0	6	0	0	0	4	0	10	0
DoubleNeg	0	174	0	0	10	1	5	0	2	0
Mono	2	7	174	0	1	108	31	30	0	19
CD141	0	0	0	167	5	0	3	0	16	1
CD1C	0	0	0	1	187	0	3	0	0	1

Table S13: PBMC2 data, Seurat

	1	2	3	4	5
pDC	175	3	8	0	6
DoubleNeg	0	171	9	0	12
Mono	1	316	38	0	17
CD141	0	0	3	161	28
CD1C	0	0	4	3	185

Table S14: PBMC2 data, TSCAN

	1	2	3	4	5
pDC	171	3	8	0	10
DoubleNeg	0	177	3	0	12
Mono	1	7	363	0	1
CD141	0	2	1	168	21
CD1C	0	2	2	0	188

Table S15: PBMC2 data, SC3

S3 Sensitivity analyses

There are various ways to set the weight matrices to reflect the partial credit or penalty a user wishes to attribute to different clustering results. The weights may be derived from prior biological knowledge such as cell lineage. When one lacks external information in constructing the weights, we may estimate it from the same data set that the clustering is performed on. For illustration purposes, the examples shown in Figure 2 and Figure S3-S5 have used this internal weight. This, however, is not the preferred method and should only be used when there is no external information on the hierarchical relationships between cell types. In general, we recommend using external public databases and biological knowledge for well-studied cell types. Below we assess the sensitivities of the proposed weight metrics to the methods of constructing weight matrices.

S3.1 Sensitivity to the number of genes used to compute the weights

When weights are computed using the same dataset that the clustering is performed on, we use the genes with the most variable expression across cell types. To be specific, we compute mean expression level within each cell type, and compute the variance of these average expression levels across cell types. Top ranked genes with the largest variances will be selected as features, and their expression levels are used to compute the weights. The number of genes used in this procedure will have some impacts on the weights, and subsequently the values of the weighted metrics.

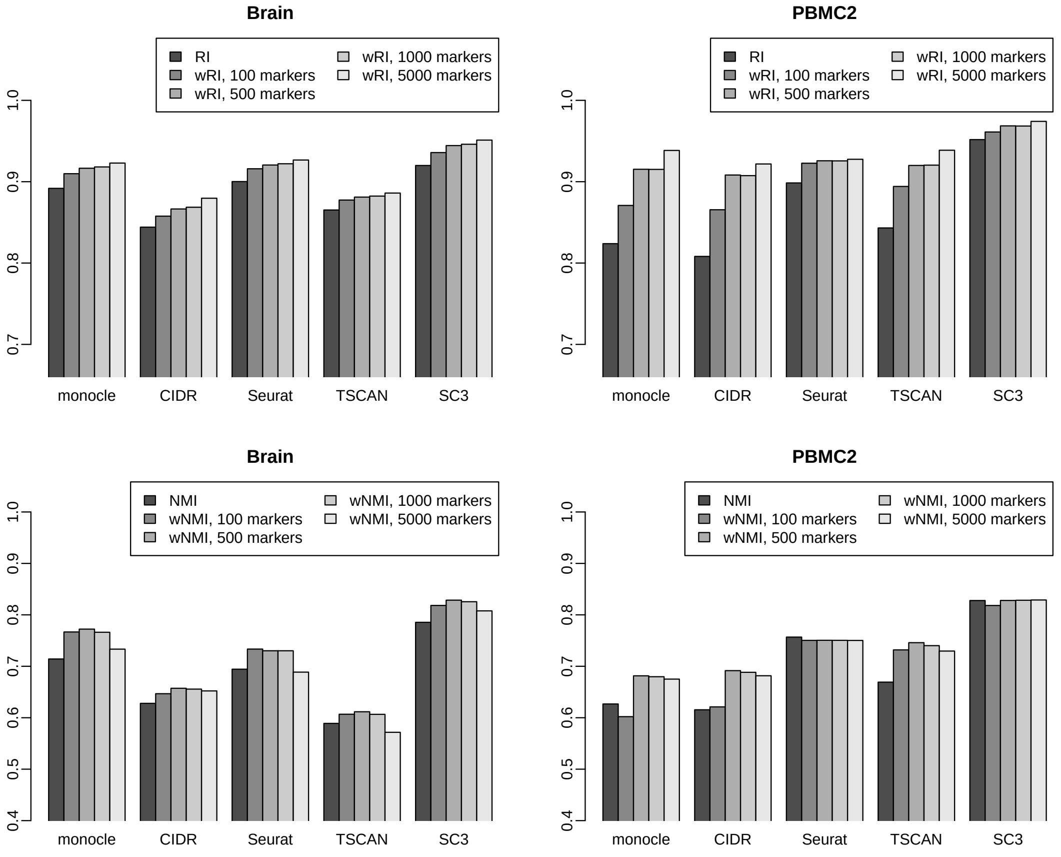

Fig. S6: wRI and wNMI from different methods using different numbers of genes to construct weight matrices.

Figure S6 shows wRI and wNMI from different methods, using different numbers of genes to construct weight matrices from the *Brain* and *PBMC2* datasets. When different number of genes are included, the wRI generally tends to give modest increase when more genes are used to compute the similarity measure. This is not surprising because when more genes are included, cells may appear more similar. On the other hand, the wNMI reaches the maximum when 500 genes are used to construct the weights for all methods. In general, we find that the relative performances among methods for the weighted metrics (wRI and wNMI) are similar regardless of the numbers of genes used. This is, however, not true when using unweighted metrics (traditional RI and NMI). For example, Seurat is significantly better than monocle, CIDR, and TSCAN in the PBMC2 dataset in terms of RI. Under wRI, these methods become rather similar when 500 or more genes are used in constructing the weights.

When using the same dataset to construct weights, in addition to select highly variable genes, another option is to directly identify genes with differential expression. Since reference cell labels are needed for traditional RI/NMI calculation, we can identify genes that demonstrate differential expression for each cell type beyond a certain false discovery rate. As discussed in the manuscript, it is critical that the choice of the reference tree/weights are made independent of the clustering methods to avoid cherry-picking.

S3.2 Sensitivity to the similarity measure

In the illustration in Figure 2 and Figure S3-S5, Pearson's correlation coefficient r is used as similarity measure for constructing the weight matrices used in the wRI. There are other choices that are qualitatively consistent. For example, note that since the power function is monotonic, r^k with other powers can also be used as similarity measures with the same orders of similarity between cell types. When k increases, the weights become closer to the identity matrix. Thus, the weighted index will reduce to the un-weighted index when k goes to infinity.

Figure S7 shows the sensitivity of wRI to different choices of similarity measure. In particular, we construct weight matrices for wRI using Pearson's correlation (r) or R^2. In general, using R^2 results in slightly lower wRI, but the relative orders of the performances from different methods remain mostly the same as using r.

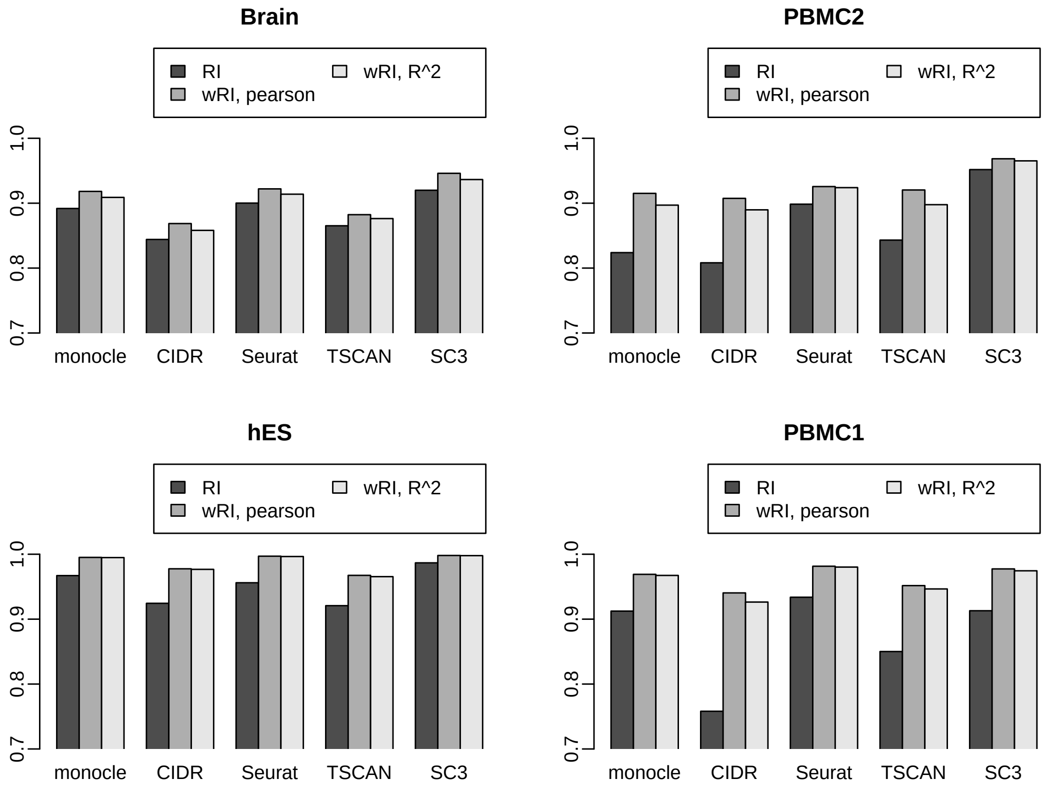

Fig. S7: wRI from different methods when weight matrix are constructed on different similarity measures.
