# Supplementary material for: Accounting for cell type hierarchy in evaluating single cell RNA-seq clustering
Source: Genome Biol. 2020 May 25;21:123. doi: 10.1186/s13059-020-02027-x (PMC7249323; doi:10.1186/s13059-020-02027-x)
Supplement: Supplementary file 2 — Additional file 2 Review history. [file 13059_2020_2027_MOESM2_ESM.docx]

Review History

**First round of review**

**Reviewer 1**

**Are you able to assess all statistics in the manuscript, including the appropriateness of statistical tests used?**

Yes: no additional statistical review is needed

**Comments to author:**

Clustering is one of the most critical steps in scRNA-seq analysis. Given that many clustering methods are available, it is important to know which method performs well. Wu et al. noted that commonly used metrics for clustering evaluation, such as Adjusted Rand Index and Normalized Mutual Information, can be misleading since these metrics do not account for the intrinsic hierarchical structure of cell types. This is a very important observation, and I am glad that the authors noticed this and proposed new metrics that can incorporate hierarchical structure naturally based on Rand Index and Normalized Mutual Information. The authors provided a nice constructed toy example that illustrated the idea, and further analyzed 4 scRNAseq datasets to show the desired performance of the proposed new metrics. The paper is concisely written. Below I list a few comments that hope to help the authors further improve the manuscript.

1. It would be helpful to briefly describe what each clustering algorithm does so that the readers can have a better understanding of the results.

2. I suggest moving weight computation into Methods as that is the most important contribution of the paper, and it should be described in the main text, not in the supplement.

3. I also think results for all 4 analyzed datasets should be included in the main text. For the the hES, Brain, and PBMC2 datasets, can you also provide confusion matrices? Confusion matrices provide information that can help interpret the results, and thus should be included.

4. Can you explain how to obtain weight matrices using the scRNAseq data that the clustering is performed on?

5. How sensitive is the weight to the choice of highly variable genes? Currently analyses used top 1000 genes. In practice, what number would you recommend?

6. The 4 datasets analyzed in the paper are all small in size. Current scRNAseq studies often have thousands of cells. To assess the practical utility of the method, I suggest evaluating additional last scale datasets that have hierarchical cell type structures. One good dataset to consider is the macaque retina scRNAseq data generated by Peng et al. (2019 Cell DOI:https://doi.org/10.1016/j.cell.2019.01.004). This dataset has a large number of cells and complex hierarchical structure of cell types. It would be a good dataset to compare different clustering algorithms with your new metrics.

7. Seurat employs Louvain's algorithm for clustering, which requires a resolution parameter. In your analysis, what resolution parameter did you use? How does the results change with different resolution parameters?

8. For Figure 2D, it seems that cluster 2 and cluster 8 both correspond to cd14.monocytes. If you use a smaller resolution, will you get one single cluster for clusters 2+8? Comparison of the confusion matrices in Figure 2D and 2E seems to suggest that Seurat clusters the cells better than SC3, because memory.t and regulatory.t are separated better in Seurat than in SC3. However, the weighted RI and weighted NMI didn't seem to differentiate Seurat and SC3 well. Can you explain why?

**Reviewer 2**

**Are you able to assess all statistics in the manuscript, including the appropriateness of statistical tests used?**

Yes: The statistics were appropriate.

**Comments to author:**

The paper makes the point that, when evaluating a clustering algorithm against a known reference partition, a researcher might ideally take into account that some mistakes are more important than others. For example, if an algorithm mistakenly assigns both a CD4 cell and a CD8 cell to the same cluster, this is less of an issue, than, say, assigning both a CD4 cell and a muscle cell to the same cluster.

To address this issue, the authors propose modifying two common metrics of cluster quality, the adjusted rand index (ARI) and the normalized mutual information (NMI). The proposed modification is to replace binary weighting (e.g., two cells are either correctly assigned to the same cluster, or they aren't) with a more generalized weighting scheme (some cell types can be weighted to be more similar to one another).

The paper is nicely written, and the weighting idea that the authors propose is sensible. On the whole, I like the paper.

However, I have two main concerns.

The first is that, although the idea is a natural one and the paper is nicely written, I am not sure that it is at the level of Genome Biology.

The second is that, arguably the most interesting part of the proposed method is how one should choose the weights. I feel that the authors did not develop this aspect as much as they could have. For example, in the case of ARI, they propose pearson correlation. This is reasonable, in the sense that it gives a number between 0 and 1, with lower numbers being more dissimilar and higher numbers being more similar. But many other metrics of association have this property, and why might we choose one over the other? As a simple example, why use r instead of R^2? I realize this might seem like a trivial thing to worry about, but I don't think it actually is, for a few reasons. The first is interpretability. If I use a binary weighting, I at least have a reasonably good sense of what that number is telling me, even if it might not be exactly what I want. And if I wanted to re-run the analysis grouping CD4 cells and CD8 cells into just "T cells" I could, and again, I'd have a reasonably good sense of what that number is telling me. However, if I weight by correlation, I'm not really sure anymore how to interpret the number I get. More to the point, if I were to run the method using r, and found out that clustering algorithm A outperforms clustering algorithm B, but then re-ran the method using R^2 and found out that clustering algorithm B outperforms clustering algorithm A, I would feel lost. The second reason, related the previous example, is the potential for abuse by researchers wanting to show that their method is "best." Without some solid guidelines on which weights should be used, researchers might simply fiddle with weighting schemes until they come out ahead.

**Authors Response**

**Point-by-point responses to the reviewers’ comments:**

Response to Reviewer 1’s comments:

Clustering is one of the most critical steps in scRNA-seq analysis. Given that many clustering methods are available, it is important to know which method performs well. Wu et al. noted that commonly used metrics for clustering evaluation, such as Adjusted Rand Index and Normalized Mutual Information, can be misleading since these metrics do not account for the intrinsic hierarchical structure of cell types. This is a very important observation, and I am glad that the authors noticed this and proposed new metrics that can incorporate hierarchical structure naturally based on Rand Index and Normalized Mutual Information. The authors provided a nice constructed toy example that illustrated the idea, and further analyzed 4 scRNAseq datasets to show the desired performance of the proposed new metrics. The paper is concisely written.

*We appreciate the positive comments from the reviewer.*

Below I list a few comments that hope to help the authors further improve the manuscript.

1.	It would be helpful to briefly describe what each clustering algorithm does so that the readers can have a better understanding of the results.

*Thanks for the comment. We have added following description of each clustering algorithm in the Results Section where these methods are mentioned (page 3-4)*

*SC3 uses a consensus matrix to summarize K-means clustering results over a series of PCA and Laplacian transformed feature matrices, followed by complete-linkage hierarchical clustering into k groups. Seurat first selects a set of highly variable genes followed by PCA dimension reduction, and then uses a graph-based approach that partitions the cell distance matrix based on the top (usually 10) principal components. It constructs a K-nearest-neighbor (KNN) graph based on Euclidean distance of the PCs, and applies modularity optimization to group cells iteratively. Monocle uses PCA to reduce dimension, often followed by further nonlinear dimension reduction by tSNE or Uniform Manifold Approximation and Projection (UMAP). The clustering is done using density peak clustering or the Louvain algorithm, which is also the default choice for modularity optimization in Seurat. CIDR performs Principal coordinate analysis on a dissimilarity matrix between imputed gene expression profiles, where imputation depends on the estimated relationship between dropout rate and gene expression level. The clustering is done on the first few principal coordinates using the R package NbClust. TSCAN first groups genes by hierarchical clustering and reduces individual gene expression to average expression of gene-clusters, which are then used to estimate PCs. It then uses model-based clustering (the R package mclust) based on multivariate normal model on the PCs.*

2.	I suggest moving weight computation into Methods as that is the most important contribution of the paper, and it should be described in the main text, not in the supplement.

*We have moved the weight computation, previously in Supplemental Materials Sections 2&3, into the main text under the subtitles of “Obtaining the weights for wRI” (page 8 the second to last paragraph) and “Obtaining the weights for wNMI” (the last paragraph of page 11).*

3.	I also think results for all 4 analyzed datasets should be included in the main text. For the the hES, Brain, and PBMC2 datasets, can you also provide confusion matrices? Confusion matrices provide information that can help interpret the results, and thus should be included.

*Thank you for the comments. As the editor indicated, if accepted this manuscript will be published as a “Short Report”, which allows no more than two figures. Thus, we cannot move the supplemental figures to the main text.*

*We have made confusion matrices for hES, Brain, and PBMC datasets, included as Section S2.2-S2.4 in the Supplemental Materials.*

4.	Can you explain how to obtain weight matrices using the scRNAseq data that the clustering is performed on?

*This was provided in the Supplemental Materials, Section 2 (now merged into the main text following the suggestion in Comment 2). We have expanded the subsection “Obtaining the weights for wRI” to describe general strategies for obtaining weights (page 7-8).*

5.	How sensitive is the weight to the choice of highly variable genes? Currently analyses used top 1000 genes. In practice, what number would you recommend?

*This is a great question. Feature selection is a very important step, not only for constructing reference cell lineage tree under our context, but also for cell clustering in general. Each cell clustering method has its own feature selection criteria, mostly based on the means and variances of the expressions. However, we first would like to point out that using the same dataset that the clustering is performed to construct the weights is only a choice when there is no external standard available. We have expanded the subsection “obtaining the weights for wRI” to reflect on the choice and strategies for the construction of weights (page 7-8).*

*We performed additional analyses using different numbers of most variable genes to construct the reference tree. The figures below summarize the wRI and wNMI using different number of genes to construct the weights for Brain and PBMC2 data. Overall, even though the numbers of genes used have some impact on the values of wRI and wNMI, the relative orders of the weighted metrics from different methods mainly remain the same. This is not true when using unweighted metrics (traditional RI and NMI). For example, Seurat is significantly better than monocle, CIDR, and TSCAN in the PBMC2 dataset in terms of RI. Under wRI, these methods become rather similar when 500 or more genes are used in constructing the weights.*

*These sensitivity analysis results and related discussions are now included in the Supplemental Materials (Section S3.1 and Figure S6).*

6.	The 4 datasets analyzed in the paper are all small in size. Current scRNAseq studies often have thousands of cells. To assess the practical utility of the method, I suggest evaluating additional last scale datasets that have hierarchical cell type structures. One good dataset to consider is the macaque retina scRNAseq data generated by Peng et al. (2019 Cell DOI:https://doi.org/10.1016/j.cell.2019.01.004). This dataset has a large number of cells and complex hierarchical structure of cell types. It would be a good dataset to compare different clustering algorithms with your new metrics.

*The PMBC1 dataset has ~4000 cells. Even though it is not as big as the most recent datasets with tens even hundreds of cells, it is not a “small” dataset.*

*We obtained the dataset suggested by the reviewer and find that reference cell types are not available, thus neither the traditional nor the proposed the metrics to compare clustering results to a gold standard could be computed.*

7.	Seurat employs Louvain's algorithm for clustering, which requires a resolution parameter. In your analysis, what resolution parameter did you use? How does the results change with different resolution parameters?

*The current Seurat results are based on the default resolution parameter, which is 0.8. We reran Seurat on the PBMC1 dataset with different resolution parameters, and then re-evaluated the clustering accuracy by traditional and proposed metrics. The figure below summarizes the findings. With larger resolution parameters, Seurat produces more clusters. In general, having more clusters tends to result in lower clustering accuracy from all metrics. The differences between the weighted and unweighted indices are rather similar though. Thus, the resolution parameter in Seurat, or the number of clusters in general, does not have a significant impact on the proposed new metrics.*

8.	For Figure 2D, it seems that cluster 2 and cluster 8 both correspond to cd14.monocytes. If you use a smaller resolution, will you get one single cluster for clusters 2+8? Comparison of the confusion matrices in Figure 2D and 2E seems to suggest that Seurat clusters the cells better than SC3, because memory.t and regulatory.t are separated better in Seurat than in SC3. However, the weighted RI and weighted NMI didn't seem to differentiate Seurat and SC3 well. Can you explain why?

*The results we showed in the Figure 2 are based on the default resolution parameter in Seurat. We tried Seurat with different resolution parameters. With lower resolution, Seurat produces fewer clusters. We were able to find a resolution parameter that produces 7 clusters. The confusion matrix for 7 clusters is shown below. It indeed puts most cd14.monocytes cells into one cluster (however, more naïve T cells are separated from their main cluster). For this result, the wRI is slighted higher than the one with 8 clusters (0.983 vs. 0.974), so is wNMI (0.933 vs. 0.922).*

cd56.nk	589	0	1	5	0	2	0

cd14.monocytes	0	587	1	0	0	0	6

b.cells	0	0	494	0	0	0	0

memory.t	0	0	0	470	1	25	3

naive.t	0	0	1	2	457	2	35

naive.cytotoxic	0	0	1	5	5	384	0

regulatory.t	2	0	0	9	148	0	337

cd4.t.helper	1	1	1	2	218	3	173

*Regarding the performances of Seurat and SC3, in terms of the wRI and wNMI measures, Seurat gave slightly higher values, and these values increase when the number of clusters is 7 by manipulating the resolution parameter. In the original result presented with 8 clusters, Seurat broke cd14.monocytes into (mostly) two clusters, whereas SC3 was able to keep them together. However, Seurat performed better in other aspects: it has a relatively pure cluster 6 (consisted primarily of 433 memory T cells) and it is able to separate these memory T-cells from a group of regulatory T/CD4 T helper cells. SC3, on the other hand, has difficulty differentiating memory T, regulatory T and CD4 T helper cells. But because these were in a lower branch, the mistakes were not penalized as much. Because both methods have their advantages and disadvantages, their mistakes balanced out and had similar overall scores.*

*In addition, we have added a paragraph in the Discussion section (second paragraph) to make it clear that the purpose of this manuscript is not to compare clustering methods, because the performance of these depends on many choices. How to make the best choice for each clustering method is beyond the scope of this manuscript.*

Response to Reviewer 2’s comments:

The paper makes the point that, when evaluating a clustering algorithm against a known reference partition, a researcher might ideally take into account that some mistakes are more important than others. For example, if an algorithm mistakenly assigns both a CD4 cell and a CD8 cell to the same cluster, this is less of an issue, than, say, assigning both a CD4 cell and a muscle cell to the same cluster.

To address this issue, the authors propose modifying two common metrics of cluster quality, the adjusted rand index (ARI) and the normalized mutual information (NMI). The proposed modification is to replace binary weighting (e.g., two cells are either correctly assigned to the same cluster, or they aren't) with a more generalized weighting scheme (some cell types can be weighted to be more similar to one another).

The paper is nicely written, and the weighting idea that the authors propose is sensible. On the whole, I like the paper.

*We appreciate the positive comments from the reviewer.*

However, I have two main concerns.

The first is that, although the idea is a natural one and the paper is nicely written, I am not sure that it is at the level of Genome Biology.

*We appreciate that the reviewer finds the idea natural and the paper nicely written. We would like to point out that, though clustering has been widely applied in genomic research since the microarray era, and numerous methods have been developed for or adapted to genomic data, the most common evaluation metrics have remained at the ones developed by computer scientists for situations involving groups without hierarchy. The idea of taking into account the hierarchical structure has not been published. The importance of the new metrics will become more apparent as our definition about cell types evolve when we gather more details in cellular profiles further divide the cell types into more subtypes.*

The second is that, arguably the most interesting part of the proposed method is how one should choose the weights. I feel that the authors did not develop this aspect as much as they could have. For example, in the case of ARI, they propose pearson correlation. This is reasonable, in the sense that it gives a number between 0 and 1, with lower numbers being more dissimilar and higher numbers being more similar. But many other metrics of association have this property, and why might we choose one over the other? As a simple example, why use r instead of R^2? I realize this might seem like a trivial thing to worry about, but I don't think it actually is, for a few reasons. The first is interpretability. If I use a binary weighting, I at least have a reasonably good sense of what that number is telling me, even if it might not be exactly what I want. And if I wanted to re-run the analysis grouping CD4 cells and CD8 cells into just "T cells" I could, and again, I'd have a reasonably good sense of what that number is telling

me. However, if I weight by correlation, I'm not really sure anymore how to interpret the number I get. More to the point, if I were to run the method using r, and found out that clustering algorithm A outperforms clustering algorithm B, but then re-ran the method using R^2 and found out that clustering algorithm B outperforms clustering algorithm A, I would feel lost.

The interpretation of the wRI is the agreement between the clustering and the reference accounting for partial agreements. The reviewer is correct that the partial agreement depends on the assignment of the weights, so does the interpretation. Any similarity matrix could be used to construct the weights. The example of using correlation coefficient is simply used for illustration. We could use R^2 and, in fact, use R^k with any k. When k is larger it resembles the unweighted version more. We completely agree that, as the reviewer points out, the orders of methods in their performance may change when different weights are used. This is the same as that the choice of any loss function could change the performance of methods. The key is to choose a reasonable loss function before evaluation instead of trying many different loss functions and pick the one that gives the most favorable results.

In many situations, the right question here is “how much of the true hierarchical cell type structure is recovered by the clustering”, or “how much the clustering agrees with the true hierarchical cell type structure”. Using an imperfect tree reference is getting an approximate answer to the right question. Compared to having the exact answer to the wrong question (how much the clustering agrees with exchangeable cell type labels), we believe that an approximate answer to the right questions is more useful to the exact answer to the wrong question, to rephrase John Tukey.

*We have performed additional sensitivity analyses on this point. The results for four datasets are summarized in the figure below. In general, using R^2 to construct similarity matrix results in slightly lower wRI, but the orders of the performances from different methods remain mostly the same. This results and related discussions are now included in the Supplemental Materials Section S3.2 and Figure S7.*

The second reason, related the previous example, is the potential for abuse by researchers wanting to show that their method is "best." Without some solid guidelines on which weights should be used, researchers might simply fiddle with weighting schemes until they come out ahead.

*The reviewer raised an important issue related to reproducible research and potential “cherry-picking”. Just as researchers could play with many tuning parameters such as the numbers of PC to use as input, the resolution parameters, the perplexity parameters to improve the performance of certain methods evaluated by RI/NMI, they could potentially play with the weight matrices as yet another dimension to make one method appear better than others. In addition, users can already fiddle with their standard by how detailed the standard they go by – whether to use only “T-cells” as one group or to further divide them into subtypes of T-cells by surface markers.*

*We have added a paragraph in the Discussion section (the third paragraph) to point out this issue and provided our recommendation.*

**Second round of review**

**Reviewer 1**

Thanks for addressing my previous concerns. The only one additional comment I have this time is on estimating the weights from the same dataset that the clustering is performed on. Since batch effect is prevalent, especially in human data, it would be helpful if the authors could comment on how batch effect is dealt with when calculating mean expression for each cell type. Would you recommend correcting batch effect in the original gene expression space and then calculate mean expression based on the corrected gene expression?
